# Incorporating electrical impedance tomography to transpulmonary pressure-guided PEEP in severe ARDS with pneumothorax and multiple cavitations: a case report

**DOI:** 10.3389/fmed.2023.1240321

**Published:** 2023-08-28

**Authors:** Qianling Wang, Longxiang Su, Jing Jiang, Na Wang, Huaiwu He, Yun Long

**Affiliations:** ^1^Department of Critical Care Medicine, State Key Laboratory of Complex Severe and Rare Diseases, Peking Union Medical College Hospital, Peking Union Medical College, Chinese Academy of Medical Science, Beijing, China; ^2^Department of Critical Care Medicine, Chongqing General Hospital, Chongqing, China

**Keywords:** positive end-expiratory pressure, transpulmonary pressure, ARDS, EIT, MRSA pneumonia

## Abstract

Pneumothorax is a potentially fatal complication in patients with acute respiratory distress syndrome (ARDS), presenting challenges in determining the optimal positive end-expiratory pressure (PEEP) level to prevent atelectasis without exacerbating the pneumothorax. This case report describes the successful application of transpulmonary pressure and electrical impedance tomography (EIT) at the bedside to guide PEEP selection in a patient with ARDS complicated by pneumothorax due to methicillin-resistant *Staphylococcus aureus* infection. By using minimal PEEP to maintain positive end-expiratory transpulmonary pressure and visualizing lung reopening with EIT, the optimal PEEP level was reaffirmed, even if traditionally considered high. The patient’s condition improved, and successful weaning from the ventilator was achieved, leading to a transfer out of the intensive care unit.

**Clinical trial registration**: https://clinicaltrials.gov/show/NCT04081142, identifier NCT04081142.

## Introduction

Pneumothorax is a potentially fatal complication in patients with acute respiratory distress syndrome (ARDS), contributing to high mortality rates. Patients with severe ARDS may necessitate higher positive end-expiratory pressure (PEEP) levels to prevent atelectasis and recruit previously collapsed alveolar units, but this approach becomes contentious when concurrent pneumothorax is present. To address this challenge, adopting an individualized approach to PEEP adjustment, which carefully balances the risk of barotrauma with the need for recruitment, may prove beneficial. Transpulmonary pressure-guided PEEP selection shown to enhance patient outcomes compared to traditional methods (1). Transpulmonary pressure is the pressure difference between airway and pleural pressure, accurately reflects lung stress, independent of the chest wall. An end-expiratory transpulmonary pressure greater than zero indicates open alveoli throughout the respiratory cycle, whereas a negative value suggests a tendency for alveolar and/or small airway collapse. However, excessively high transpulmonary pressure can lead to alveolar overdistension, exacerbating pneumothorax. In severe ARDS complicated by pneumothorax, precise PEEP adjustment becomes particularly important. While esophageal pressure serves as a surrogate for pleural pressure and offers a relatively less invasive and reliable method for obtaining transpulmonary pressure, factors like mediastinal weight, abdominal pressure, and esophageal balloon positioning may influence measurements (2). Therefore, ensuring the accuracy of transpulmonary pressure-guided PEEP selection through alternative methods is necessary.

Electrical Impedance Tomography (EIT) is a non-invasive, radiation-free bedside imaging technique that provides real-time ventilation monitoring by estimating changes in lung resistivity during respiration, reflecting alterations in intrapulmonary gas volume and conductivity (3). The real-time monitoring provided by EIT offers a valuable solution to address concerns regarding the accuracy of positive PEEP solely determined by esophageal pressure. A recent brief report by Slobod et al. (4) proposed a novel approach to personalize positive PEEP for intubated hypoxemic patients undergoing pressure support ventilation. Their method involves integrating transpulmonary pressure into EIT-based regional compliance calculations. Although promising, further validation is required to ascertain whether this approach indeed improves patient outcomes. In our current case, we propose an alternative approach that integrates EIT-based regional ventilation information into the PEEP adjustment guided by transpulmonary pressure for a patient with ARDS complicated by severe atelectasis and pneumothorax. Our aim is to achieve a more precise and personalized method to address the specific challenges for this patient, potentially leading to improved clinical outcomes.

## Case presentation

**Figure 1:**
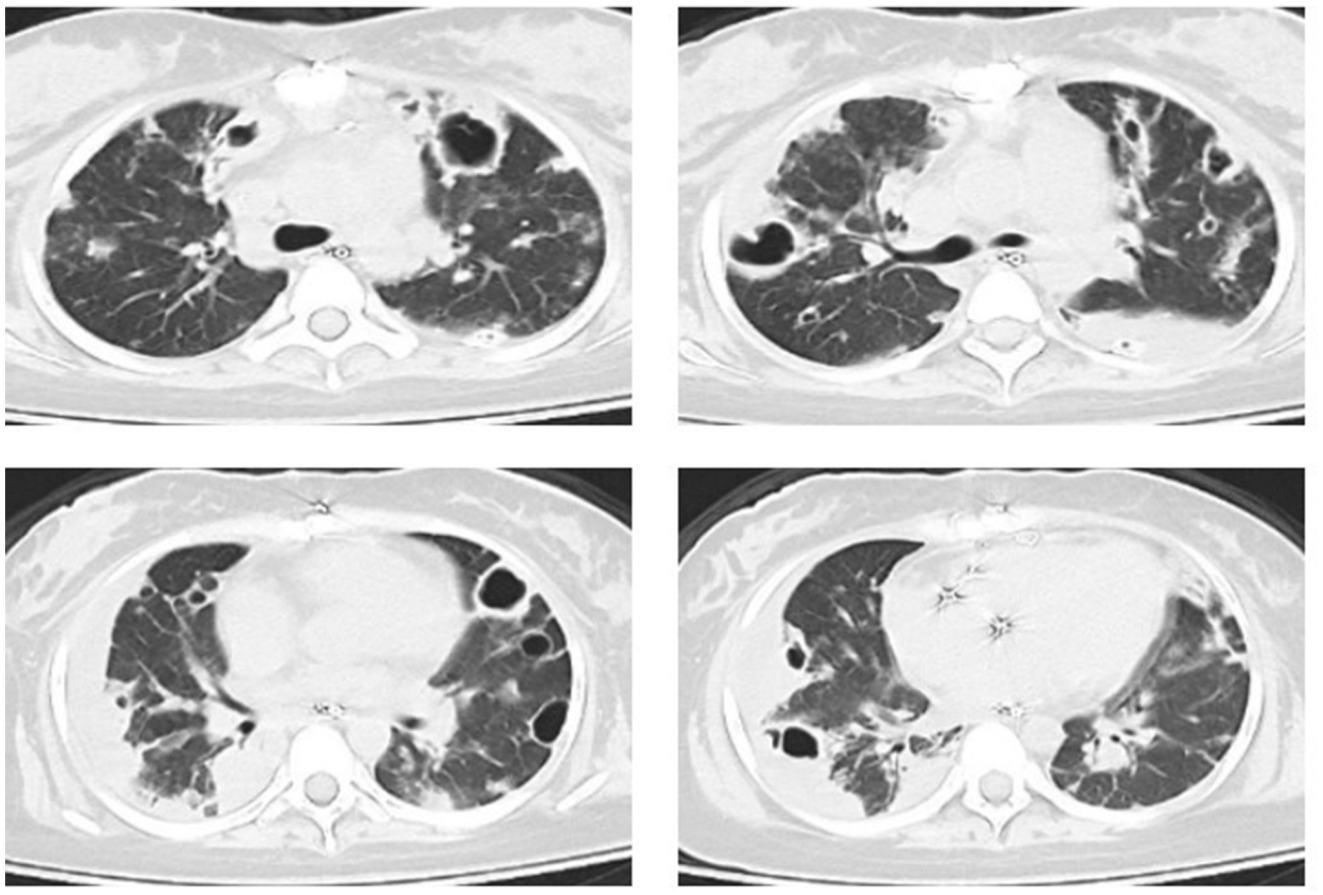
CT scan showed multiple cavitary infiltrates in both lungs on admission.

An 18-year-old female patient was transferred from another hospital to our emergency department with a critical condition of respiratory failure and septic shock, which resulted from the dissemination of methicillin-resistant *Staphylococcus aureus* (MRSA) in her bloodstream, originating from septic arthritis of the left hip. The patient had no history of intravenous drug use or recent hospitalization. During the examination, the patient presented with a heart rate of 112 beats per minute, a temperature of 38.2°C, and a respiratory rate of 22 breaths per minute. Her mean arterial pressure was maintained within the range of 65 to 75 mmHg with the administration of norepinephrine at 0.21 μg/kg/min. The patient had been intubated prior to admission and required mechanical ventilation in pressure support mode, with a pressure support level of 12 cmH2O, PEEP of 6 cmH2O, and an inspired oxygen fraction (FiO2) of 0.4, resulting in a partial pressure of arterial oxygen to inspired oxygen fraction (PaO2/FiO2) ratio of 215 mmHg. A chest computed tomography (CT) scan conducted after her admission revealed multifocal pulmonary opacities and cavitations (Figure 1). Subsequent cardiac ultrasound identified a tricuspid valve vegetation formation measuring 14 mm × 12 mm, along with tricuspid valve leaflet destruction, leading to severe regurgitation, raising concerns for infective endocarditis. The patient underwent tricuspid valve replacement surgery the following day and was immediately transferred to the intensive care unit (ICU).

**Figure 2:**
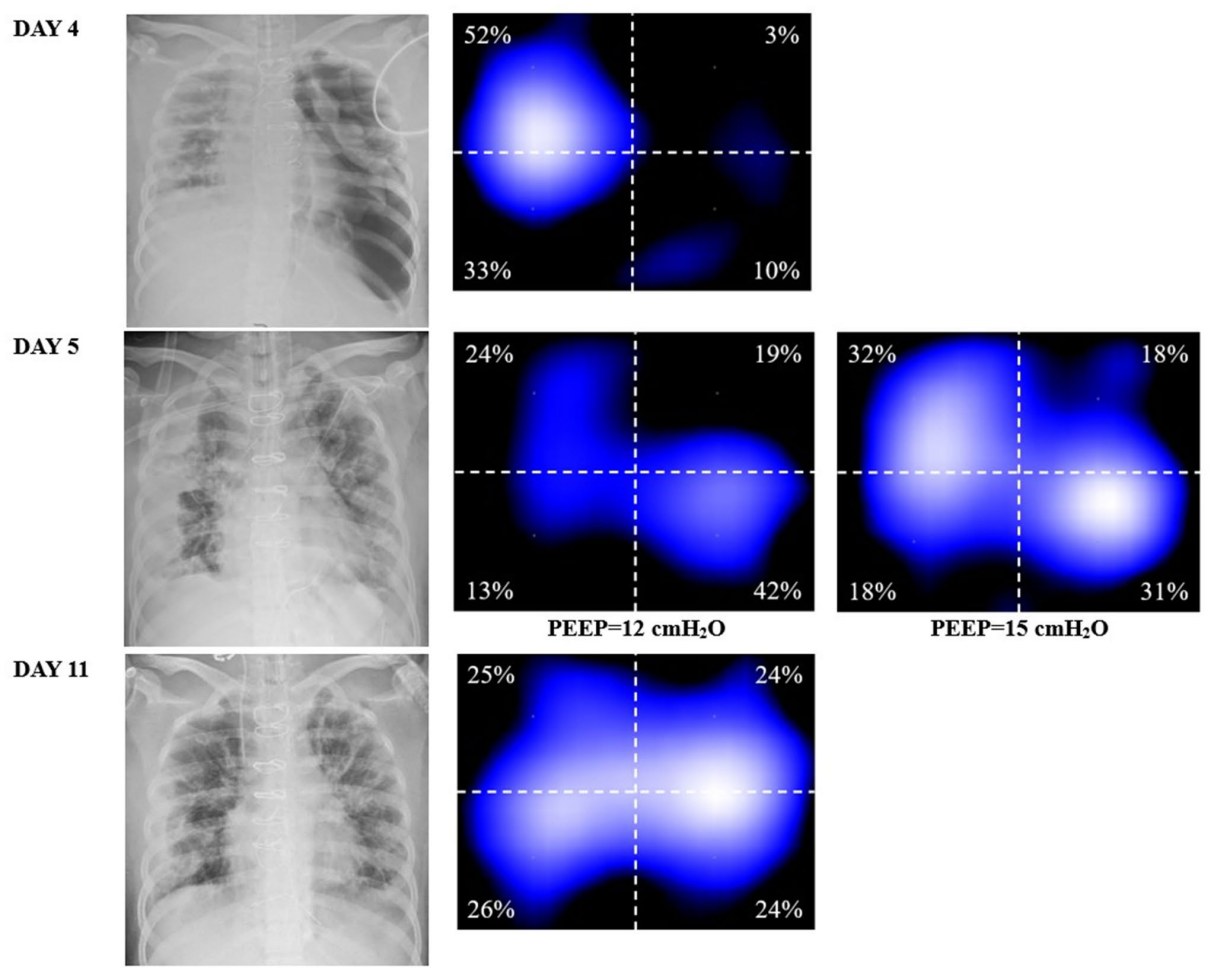
DAY 4, the chest X-ray indicating tension pneumothorax and the corresponding EIT ventilation image, showing a significant decrease in ventilation in the left lung. DAY 5, chest X-ray (at a PEEP of 6 cmH2O) shows left lung reopening after transthoracic drainage, but right lung atelectasis. At a PEEP of 15 cmH2O, the right lung was better ventilated compared to that at 12 cmH2O (well ventilated regions in light blue in EIT). DAY 11, improvement in exudation and atelectasis, the left side chest drain has been removed, and the homogeneity of ventilation in both lungs has been improved compared to the previous status.

On DAY 2 after admission to the ICU, a chest X-ray revealed evident exudation and consolidation in both lungs, particularly in the dependent areas. Further ultrasound imaging indicated no significant abnormalities in cardiac function. The patient was under volume-controlled ventilation with a tidal volume (VT) of 5.8 mL/kg predicted bodyweight, a PEEP level of 6 cm H2O, a respiratory rate of 22 breaths/min, and a plateau pressure of 26 cmH2O for lung-protective ventilation. Her PaO2 was 91 mm Hg on an FiO2 of 0.5 (Table 1). Prone position and anti-infective treatment were also administered. On Day 4 after ICU admission, she developed a left-sided tension pneumothorax (Figure 2) and received immediate emergency decompression with a chest drain. On Day 5 after ICU admission, her oxygenation significantly deteriorated (PaO2/FiO2 ratio decreased from 182 to 98). The chest X-ray displayed diffuse exudation in both lungs and significant atelectasis on the right side (Figure 2). Additionally, the left-side thoracic drainage showed ongoing air leaks. In an effort to minimize the risk of alveolar hyperinflation, the tidal volume was further reduced to 3 mL/kg predicted bodyweight to lower the plateau pressure. Additional respiratory parameters of the patient are presented in Table 1. Despite these efforts, the air leaks persisted, and oxygenation did not improve, prompting the need for more precise PEEP settings.

**Table 1:** Respiratory parameters of the patient during ICU admission.

Date	DAY 2	DAY 5	DAY 6	DAY 7	DAY 8	DAY 9	DAY 10	DAY 11
Tidal volume (ml/kg predicted bodyweight)	5.8	3	3	3.5	4	4	6	6
Respiratory rate (breaths per minute)	22	34	32	30	28	28	18	18
PaCO2 (mmHg)	39	58	56	49	48	45	42	40
Plateau pressure (cmH2O)	24	25	25	25	24	23	20	20
PEEP (cmH2O)	6	15	15	12	12	12	9	9
End-expiratory transpulmonary (cmH2O)	NA	0.7	0.9	1.0	0.9	0.9	0.6	0.8
Ventilatory mode	VC	VC	VC	VC	VC	VC	VC	VC
PaO2/FiO2	182	98	115	122	167	191	216	230

PaCO2 denotes arterial partial pressure of carbon dioxide; PEEP denotes positive end-expiratory pressure; PaO2/FiO2 denotes partial pressure of arterial oxygen to inspired oxygen fraction; DAY 2 denotes the DAY 2 of ICU admission; VC denotes volume control; NA denotes not available.

As transpulmonary pressure eliminates the interference of pleural and abdominal pressures, providing a more accurate method for determining the optimal PEEP compared to plateau pressure, and EIT allows real-time bedside visualization of the effects of different PEEP levels determined by transpulmonary pressure on ventilation. Therefore, we employed esophageal pressure combined with EIT to select the optimal PEEP, aiming to avoid lung injury caused by alveolar overdistension while preventing atelectasis and recruiting previously collapsed alveolar units. She remained deeply sedated, and a continuous infusion of neuromuscular blocking agents was administered to ensure controlled ventilation and prevent spontaneous breathing efforts. The protocol for esophageal pressure measurement followed the methods described in our previous study (5). Setting the PEEP at 12 cmH2O resulted in an end-expiratory esophageal pressure of 16.9 cmH2O, corresponding to an end-expiratory transpulmonary pressure of −4.9 cmH2O. However, when the PEEP was increased to 15 cmH2O, the end-expiratory esophageal pressure reduced to 15.7 cmH2O, and the end-expiratory transpulmonary pressure became positive, reaching 0.7 cmH2O. Therefore, a PEEP of 15 cmH2O was considered the minimum PEEP required to effectively maintain alveolar recruitment and prevent atelectasis.

Remarkably, within 10 min of increasing the PEEP from 12 to 15 cmH2O (Figure 2), significant recruitment in the right lung was observed without an increase in the volume of air leakage. Ventilation distribution was closely monitored for the following 6 h to ensure no further pneumothorax occurred. Concurrently, end-expiratory lung impedance increased during the EIT measurement period, confirming the positive response to the adjusted PEEP. For EIT measurements, an EIT electrode belt with 16 electrodes was positioned around the thorax in the fourth to fifth intercostal space, with one reference electrode placed on the patient’s abdomen using the PulmoVista 500 system by Dräger Medical, Lübeck, Germany.

From day 6 of ICU admission, no additional air leaks were recorded. Arterial oxygenation improved, and the chest X-ray showed a reduction in atelectasis and exudation. On day 11 of ICU admission, the thoracic drainage tube was safely removed, and the ventilator mode was transitioned from volume-controlled ventilation to pressure-supported ventilation. Gradually, her oxygenation improved, and a lung CT on day 19 of ICU admission revealed significant absorption of bilateral pulmonary infiltrates and cavitation, with no signs of pneumothorax. The patient was successfully extubated the following day and subsequently transitioned to high-flow nasal oxygen therapy. Eventually, the patient was discharged from the ICU on the 25th day of admission.

## Discussion

Severe ARDS caused by MRSA bloodstream infection, complicated by multiple cavitation and pneumothorax, is associated with a very high rate of morbidity and mortality. In this case, we achieved more accurate PEEP in a mechanically ventilated patient using a combination of transpulmonary pressure and EIT. As a result, the patient gradually recovered, was successfully weaned from the ventilator, and transferred out of the ICU.

PEEP plays a crucial role in preventing atelectasis and reducing atelectrauma caused by periodic recruitment and decruitment. In cases of severe ARDS, higher levels of PEEP may be required to achieve the treatment targets (6). However, in patients complicated by pneumothorax and cavitation, setting higher PEEP levels can be a dilemma as it may induce or worsen pneumothorax in such individuals (7, 8). Previous studies have analyzed the association between airway pressure and barotrauma risk in patients with acute lung injury (ALI)/ARDS. Eisner et al. (9) retrospectively analyzed a cohort of 718 patients without baseline barotrauma and found that higher PEEP was associated with an increased risk of early barotrauma. Similarly, a study in patients with ARDS due to COVID-19 also reported similar results (10). The underlying reason could be that excessive PEEP may lead to an increased transpulmonary pressure, potentially causing barotrauma. However, it is crucial to recognize that higher PEEP levels is not an absolute contraindication for pneumothorax, as this does not consistently signify excessive transpulmonary pressure, which is the primary determinant contributing to alveolar overdistension. A systematic review and meta-analysis have suggested that the occurrence of pneumothorax is not significantly related to high PEEP (11). Therefore, in severe ARDS with pneumothorax and multiple cavitations, achieving an appropriate PEEP setting becomes challenging due to the need to carefully balance alveolar recruitment and overdistension.

If we have to make a choice between low PEEP and high PEEP for this patient, we must carefully consider the benefits and potential harms of each approach. High PEEP offers the advantage of avoiding atelectrauma, which is caused by alveolar collapse associated with low end-expiratory lung volumes and injuries resulting from mechanical forces involved in repeated opening and closing of small bronchioles and alveoli during tidal ventilation (12). By maintaining alveolar opening throughout the respiratory cycle, high PEEP increases functional residual capacity and lung compliance, reducing the risk of atelectrauma. However, the high PEEP carries the disadvantage of potentially inducing barotrauma due to increased transpulmonary pressure and alveolar overdistension, which could worsen pneumothorax – a concerning complication in patients with pneumothorax and multiple cavitations. The advantages and disadvantages of Low PEEP are opposite to those of High PEEP. On the DAY 5 of ICU admission, the patient experienced a significant exacerbation of oxygenation due to the emergence of severe atelectasis, alongside existing pneumothorax and multiple cavitations. Therefore, we believe that the optimal PEEP for this patient is the minimum level required to keep the alveoli open even at end-expiration. During controlled ventilation, transpulmonary pressure is calculated as the difference between PEEP (a surrogate for airway pressure) and esophageal pressure (a surrogate for pleural pressure) at end-expiration (13). A transpulmonary pressure greater than zero indicates that the alveoli remain open throughout the respiratory cycle (14). A previous study suggested that mortality might be reduced when PEEP titration achieves end-expiratory transpulmonary pressure near 0 cm H2O in patients with ALI or ARDS (1). In this case, a PEEP of no less than 15 was required to achieve a positive end-expiratory transpulmonary pressure, which may be considered relatively high according to traditional standards.

Given the concerns about the high PEEP and the accuracy of the transpulmonary pressure measurement, we employ a more intuitive way to prove the reliability of this PEEP. EIT is a non-invasive and radiation-free clinical imaging tool used to monitor ventilation distribution in real-time and at the bedside (15). It provides continuous image monitoring, helping optimize mechanical ventilation settings, and detect complications such as decruitment and pneumothorax. EIT’s ability to detect even small pneumothoraces of 20 mL in real-time has been demonstrated previously (16). With the help of EIT, we confirmed a substantial improvement in ventilation distribution on the right lung after increasing PEEP from 12 to 15 cmH2O. The images showed a more homogeneous distribution between the lung areas. This evidence allowed us to titrate mechanical ventilation parameters more accurately and individually, overcoming concerns associated with the use of ‘high PEEP’ in ARDS complicated by pneumothorax.

Clinical practice can be challenging for clinicians, especially when dealing with non-standard patients and conflicting treatment options, as often seen in critically ill patients. The choice of the best option in such situations is crucial. In severe ARDS, a single approach to determining the optimal PEEP may have limitations, and no single method has been shown to improve clinical outcomes significantly compared to others (12). Combining different techniques, such as EIT and esophageal pressure, to determine the optimal PEEP more accurately could lead to improved clinical outcomes or provide solutions to clinical dilemmas.

In conclusion, pneumothorax is a life-threatening complication of severe ARDS, and individualizing PEEP is crucial to achieve alveolar recruitment while avoiding alveolar overdistension or exacerbation of pneumothorax. We presented a novel idea of incorporating regional ventilation information provided by EIT as a safety measure for severe ARDS complicated with pneumothorax and multiple cavitations after transpulmonary pressure-guided PEEP selection. In the complex clinical settings of mechanical ventilation, a more precise approach could yield benefits by involving the combination of various techniques rather than relying solely on a single approach in the future.

## Data availability statement

The raw data supporting the conclusions of this article will be made available by the authors, without undue reservation.

## Ethics statement

Written informed consent was obtained from the individual(s) for the publication of any potentially identifiable images or data included in this article.

## Author contributions

QW and HH prepared the final copy of the manuscript and EIT images. LS, JJ, and NW performed the EIT examination. QW obtained patient’s consent. HH and YL edited and revised the manuscript. All authors contributed to the article and approved the submitted version.

## Funding

This work was supported by the National High-Level Hospital Clinical Research Funding (2022-PUMCH-D-005, 2022-PUMCH-B-115).

## Conflict of interest

The authors declare that the research was conducted in the absence of any commercial or financial relationships that could be construed as a potential conflict of interest.

## Publisher’s note

All claims expressed in this article are solely those of the authors and do not necessarily represent those of their affiliated organizations, or those of the publisher, the editors and the reviewers. Any product that may be evaluated in this article, or claim that may be made by its manufacturer, is not guaranteed or endorsed by the publisher.
